# LOXL1 promotes tumor cell malignancy and restricts CD8 + T cell infiltration in colorectal cancer

**DOI:** 10.1007/s10565-024-09840-1

**Published:** 2024-01-25

**Authors:** Chenxi Li, Siqi Chen, Xiaona Fang, Yaqing Du, Xin-Yuan Guan, Runhua Lin, Liang Xu, Ping Lan, Qian Yan

**Affiliations:** 1 Guangdong Provincial Key Laboratory of Colorectal and Pelvic Floor Diseases, Guangdong Institute of Gastroenterology, The Sixth Affiliated Hospital, Sun Yat-Sen University, Room 703, Building No. 3, 26 Yuancun ERheng Road, Guangzhou, 510655 China; 2 Department of General Surgery (Colorectal Surgery), The Sixth Affiliated Hospital, Sun Yat-Sen University, Room 703, Building No. 3, 26 Yuancun ERheng Road, Guangzhou, 510655 China; 3 Biomedical Innovation Center, The Sixth Affiliated Hospital, Sun Yat-Sen University, Room 703, Building No. 3, 26 Yuancun ERheng Road, Guangzhou, 510655 China; 4 Sun Yat-sen University Cancer Center, State Key Laboratory of Oncology in South China, Collaborative Innovation Center for Cancer Medicine, Sun Yat-sen University, Guangzhou, China; 5 Department of Pediatric Oncology, Sun Yat-sen University Cancer Center, Guangzhou, China; 6 Institute of Basic Medical Sciences, School of Life Sciences and Biopharmaceuticals, Guangdong Pharmaceutical University, Guangzhou, China; 7 Department of Clinical Oncology, The University of Hong Kong, Hong Kong, China; 8 State Key Laboratory for Liver Research, The University of Hong Kong, Hong Kong, China; 9 Department of Pathology, Shantou University Medical College, Shantou, China; 10 Department of Pathology, The Sixth Affiliated Hospital, Sun Yat-sen University, Guangzhou, China; 11 State Key Laboratory of Oncology in South China, Sun Yat-sen University, Guangzhou, China

**Keywords:** Colorectal cancer, Lysyl oxidase like 1, Prognosis, CD8 + T cell, Immune cell infiltration, Immunotherapy

## Abstract

**Background:**

Colorectal cancer (CRC) is a leading cause of cancer mortality globally. Lymph node metastasis and immunosuppression are main factors of poor prognosis in CRC patients. Lysyl oxidase like 1 (LOXL1), part of the lysyl oxidase (LOX) family, plays a yet unclear role in CRC. This study aimed to identify effective biomarkers predictive of prognosis and efficacy of immunotherapy in CRC patients, and to elucidate the prognostic value, clinical relevance, functional and molecular features, and immunotherapy predictive role of LOXL1 in CRC and pan-cancer.

**Methods:**

Weighted gene co-expression network analysis (WGCNA) was employed to explore gene modules related to tumor metastasis and CD8 + T cell infiltration. LOXL1 emerged as a hub gene through differential gene expression and survival analysis. The molecular signatures, functional roles, and immunological characteristics affected by LOXL1 were analyzed in multiple CRC cohorts, cell lines and clinical specimens. Additionally, LOXL1's potential as an immunotherapy response indicator was assessed, along with its role in pan-cancer.

**Results:**

Turquoise module in WGCNA analysis was identified as the hub module associated with lymph node metastasis and CD8 + T cell infiltration. Aberrant elevated LOXL1 expression was observed in CRC and correlated with poorer differentiation status and prognosis. Molecular and immunological characterization found that LOXL1 might mediate epithelial-mesenchymal transition (EMT) process and immunosuppressive phenotypes of CRC. Functional study found that LOXL1 enhanced tumor cell proliferation, migration and invasion. Moreover, high LOXL1 levels corresponded to reduced CD8 + T cell infiltration and predicted poor clinical outcomes of immunotherapy. Similar trends were also observed at the pan-cancer level.

**Conclusions:**

Our findings underscore the critical role of LOXL1 in modulating both malignancy and immunosuppression in CRC. This positions LOXL1 as a promising biomarker for predicting prognosis and the response to immunotherapy in CRC patients.

**Graphical Abstract:**

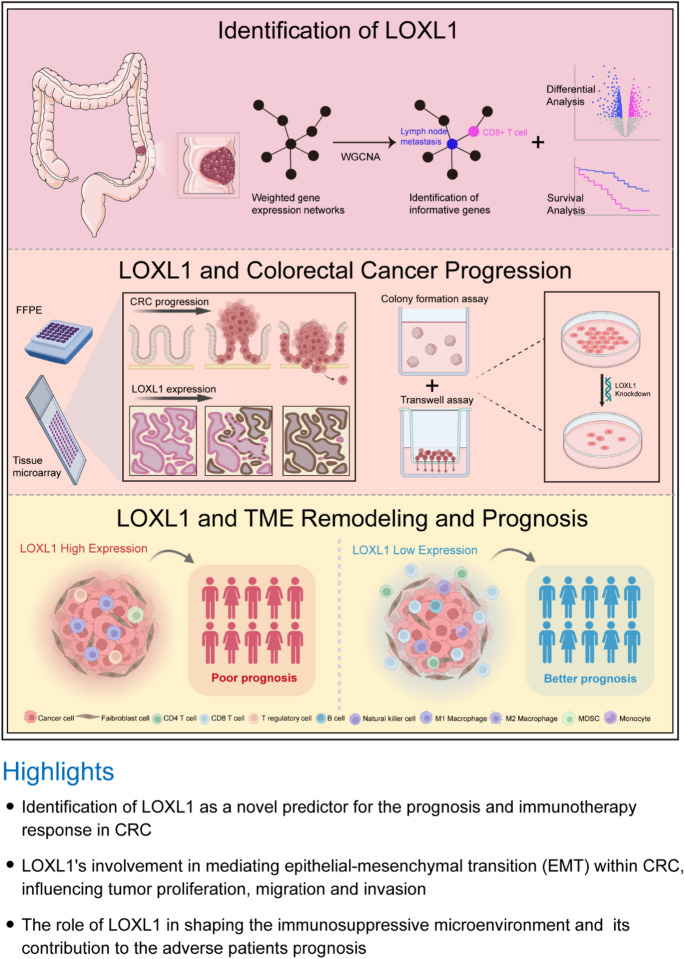

**Supplementary Information:**

The online version contains supplementary material available at 10.1007/s10565-024-09840-1.

## Introduction

Colorectal cancer (CRC) stands as a major health threat, ranking second in global cancer mortality (Dekker et al. 2019). About 900,000 people die from CRC each year, accounting for about 10% of all cancer-related deaths (Bray et al. 2018). Despite advancements in detection and therapy, late-stage CRC prognosis remains poor (Miller et al. 2016). Better understanding of CRC pathogenesis will definitely help to identify prognostic biomarkers and therapeutic targets that are essential for development of new treatment strategies.

Cancer immunotherapy (Pardoll 2012), especially immune checkpoint blockade (ICB), has shown unprecedented clinical benefits across multiple types of solid tumors (Omar and Tolba 2019; Topalian et al. 2012; Wolchok et al. 2017). Recently, therapies targeting PD-1 and PD-L1 have demonstrated efficacy in CRC (Binnewies et al. 2018; Yamamoto et al. 2017). However, only a minority of CRC patients respond to these treatments. As the major effectors of cellular adaptive immune response, CD8 + T cells are pivotal in eradicating cancer and immune surveillance (Lu et al. 2019a). Accumulating research reveals a consistent association between the presence of tumor-infiltrating CD8 + T cells and the clinical efficacy of ICB therapies in cancers, including CRC, esophageal cancer (EC), pancreatic cancer (PCA), and non-small cell lung cancer (NSCLC) (Matsumoto et al. 2016; Huang et al. 2018; Spranger et al. 2015). In CRC, the abundance and functionality of CD8 + T cells vary across different stages and subtypes. For example, advanced stages and lymph node metastasis are often characterized by reduced CD8 + T cell densities, contrasting with early-stage CRC (Trajkovski et al. 2018). Factors contributing to this disparity include persistent antigen stimulation, heightened immune checkpoint activity, and an immunosuppressive tumor microenvironment (TME) rich in stromal components, immune cells, and cytokines. These elements collectively diminish CD8 + T cell infiltration and functionality, facilitating immune escape and worsening patient outcomes (Galon and Bruni 2020). Although recent research has identified numerous biomarkers predictive of immunotherapy responses and prognosis in CRC patients, few studies have focused on the association between tumor intrinsic molecular signatures and the extrinsic microenvironment regulated by these biomarkers.

This study aims to discover prognostic biomarkers linked to immunotherapy benefits in CRC, and explore its biological functions in remodeling TME in CRC. Lysyl oxidase like 1 (LOXL1), a copper-dependent amine oxidase (Xiao and Ge 2012), is implicated in various cancers, influencing cell proliferation and metastasis through extracellular matrix (ECM) remodeling (Pez et al. 2011; Akiri et al. 2003). In gastric cancer (GC), upregulation of LOXL1 correlates with poorer differentiation, lymph node metastasis, and unfavorable prognosis (Kasashima et al. 2018). Additionally, its association with chemotherapy resistance is also noted in lung cancer and pancreatic ductal carcinoma (PDC) (Zhang et al. 2014; Calvé et al. 2016). However, much remains unknown about the role of LOXL1 in CRC. Our study elucidates LOXL1's function in shaping CRC's molecular landscape and its immunosuppressive TME, offering a new therapeutic target and prognostic indicator for CRC.

## Materials and methods

### Clinical specimens and cell lines

Our study included 208 CRC specimens and corresponding healthy tissues, collected from surgical patients at Sun Yat-sen University's Sixth Affiliated Hospital, Guangzhou, China (Table S1). Informed consent was obtained, and ethical approval was granted by the hospital's Institutional Review Board. This study employed various human CRC cell lines, namely LOVO, DLD1, SW480, SW620, THC8307, RKO, HCT15, HCT116, CT26, and MC38. Authentication of these cell lines was ensured through STR DNA profiling analysis. Cell culture conditions included high-glucose Dulbecco’s modified Eagle medium (Gibco BRL, Grand Island, NY) with a supplement of 10% fetal bovine serum (Gibco BRL) and 1% penicillin/streptomycin, maintained at 37 °C and 5% CO2 in a humidified atmosphere.

### Data acquisition and preprocessing

Gene Expression Omnibus (GEO) data was sourced from GEO website, encompassing normalized microarray data sets (GSE39582, GSE17536, GSE161158, GSE29621) and corresponding clinical details, using the “GEOquery” R package. In the preprocessing stage, probe IDs were mapped to their corresponding gene symbols based on the platform's annotation files specific to each microarray platform. When multiple probes corresponded to the same gene symbol, we selected the probe with the highest expression value to represent the final gene expression for that gene. To further refine the data quality, we conducted a quality check to identify and exclude outliers. This was followed by filtering to remove low-expressed genes, thereby reducing noise and enhancing the data quality. Lastly, we performed a Log2 transformation to normalize the data distribution, facilitating more accurate subsequent analyses.

For The Cancer Genome Atlas (TCGA) data, retrieved from TCGA portal, RNA-Sequencing data of CRC samples (41 normal, 478 tumor) and detailed clinicopathological data were standardized using the “TCGAbiolinks” R package. During preprocessing, we first normalized the RNA-Seq data to transcripts per kilobase million (TPM) to standardize the data and enable quantitative comparisons across samples. In cases of duplicate ENSEMBL IDs, we retained the ID corresponding to the highest gene expression value. Subsequently, we excluded genes with low expression value to maintain the integrity of our data.

### WGCNA construction and analysis

Utilizing GSE39582 microarray data and associated pathological information, we conducted a weighted gene co-expression network analysis. This analysis aimed to identify gene modules associated with the pathologic N stage (pN stage) and CD8 + T cell infiltration using R package “WGCNA” (Zhang and Horvath 2005). After excluding genes with minimal expression (expression value ≤ 1) from the initial pool of 19,028 genes, we narrowed down our selection based on their variance across samples. This process involved choosing the top 25% of genes exhibiting the highest variance, resulting in a set of 5,216 genes for subsequent WGCNA analysis. We firstly calculated Pearson correlation coefficients and constructed an adjacency matrix. This matrix was set with a soft thresholding power of 4 and subsequently transformed into a topological overlap matrix (TOM), grouping genes with similar expression patterns via dynamic tree-cutting method. 13 modules were identified by setting the minimum module size at 30 and the merged cut height at 0.25. Among these, exhibiting the strongest correlation with the clinical traits of interest, was identified as the hub module. This correlation was determined by assessing the Pearson correlation coefficients between gene modules and clinical traits. Gene Ontology (GO) and Kyoto Encyclopedia of Genes and Genomes (KEGG) analyses of this module's genes used “clusterProfiler” package in R, with a false discovery rate (FDR) cutoff < 0.05.

### Identification of candidate biomarkers

In the turquoise module, 134 genes were identified as hub genes, determined by module membership (|MM|> 0.8) and gene significance (|GS|> 0.2). We applied the “limma” R package for differential expression analysis between 19 para-tumor and 123 tumor tissues in the GSE39582 cohort, setting stringent thresholds (|log2FC|> 1.5, FDR < 0.01). The intersections between hub genes yielded by WGCNA and the up-regulated DEGs were taken for further examination. Kaplan–Meier survival analyses, using “survminer” R package, identified candidate biomarkers with prognostic value. This included determining optimal cutoff points for the 23 genes including LOXL1, through the same package. The cutoff values obtained from the KM analysis were consistently applied to the Cox regression analysis for categorizing groups. A threshold of *P* < 0.05 was considered statistically significant.

### Nomogram construction and validation

We assessed the independent prognostic value of these candidate genes through univariate and multivariate Cox regression analyses using the “survival” R package. Results visualization employed the “forestplot” R package. A predictive nomogram for overall survival (1-, 3-, and 5-year) was developed based on multivariate analysis results using the “rms” R package, with calibration curves assessing nomogram effectiveness.

### Western blotting and immunohistochemistry (IHC)

Western blot and IHC staining followed established protocols (Yan et al. 2021). IHC scoring utilized image pro plus software, analyzing images to determine IOD/Area or IOD Sum values. Antibodies used included LOXL1 (1:1000, PA5-79,609, Invitrogen), β-tubulin (1:1000, sc-166729, Santa Cruz) for western blotting, and LOXL1 (1:500), CD8 (ZA-0508, ZSGB-Bio) for IHC.

### RNA extraction and quantitative real-time PCR (qRT-PCR)

RNA was extracted using the TRIzol method (Invitrogen), with cDNA synthesized using the High Fidelity cDNA Synthesis Kit (Roche). qRT-PCR utilized the SYBR Green PCR Kit (Applied Biosystems) with specific primers (Table S2).

### In vitro functional assays

Cell growth assessment utilized CCK8 assays. Foci formation assays measured anchorage-dependent proliferation, seeding 1 × 103 cells in a 6-well plate, with colonies counted post two-week incubation. Soft agar colony formation assessed anchorage-independent growth. Migration assays involved serum-free DMEM in culture inserts with 8 mm filters (Corning), with 10% FBS medium below the insert. Migrated cells were counted after 48 h.

### Tumor mutation burden assessment

Somatic mutation data of the TCGA-COAD cohort from the GDC portal was organized in Mutation Annotation Format (maf). Tumor mutation burden (TMB) calculation included non-synonymous mutations, with 38 Mb as the estimated exome size. Gene mutation patterns and frequencies were analyzed and visualized using the “maftool” R package.

### Immune cell infiltration level estimation, biological function, pathway enrichment analysis

Tumor-infiltrating immune cells in GSE17536 patients were estimated using “CIBERSORT” with the “LM22” signature matrix. Biological function and pathway enrichment analyses employed gene set variation analysis (GSVA), gene set enrichment analysis (GSEA), and single sample gene set enrichment analysis (ssGSEA). The “GSVA” R package implemented GSVA, while “clusterProfiler” performed GSEA, using “h.all.v7.5.1.symbols” and “c2.cp.kegg.v7.5.1.symbols” as reference gene sets. ssGSEA quantified enrichment scores of 25 gene sets via “GSVA”. For analyses of the CIBERSORT, GSEA, GSVA, and ssGSEA, we firstly arranged the samples in descending order based on LOXL1 expression levels, and then categorized the initial 100 cases as the high-expression group and the last 100 cases as the low-expression group. The only exception was the GSE17536 cohort comprising 177 samples. We categorized the top or last 50 cases for high or low expression groups. The 25 tumor and immune-associated gene sets were collected from diverse resources through a manually extensive literature search and used as reference gene sets (Ayers et al. 2017; Lu et al. 2019b; Jiang et al. 2018; Charoentong et al. 2017) (Table S3).

### Prediction of immunotherapeutic response

Potential responses to ICB therapy were deduced using tumor immune dysfunction and exclusion (TIDE) scores and immunophenoscores (IPS), as previously described (Jiang et al. 2018; Charoentong et al. 2017). Lower TIDE or higher IPS indicated better immunotherapy response. The IMvigor210 cohort data, from patients treated with anti‐PD‐L1 agents, were sourced from IMvigor210CoreBiologies website and processed with the “IMvigor210CoreBiologies” R package.

### Statistical analysis

All statistical analyses and visualizations utilized RStudio (version 4.1.2). Student’s t-test or ANOVA assessed normally distributed variables, while chi-square tests compared categorical data. Variable correlations used Pearson or Spearman analysis based on data distribution, with statistical significance defined at *P* < 0.05 (^*^P < 0.05, ^**^P < 0.01, ^***^P < 0.001, ^****^P < 0.0001).

## Results

### Construction of WGCNA and identification of key modules

**Fig. 1:**
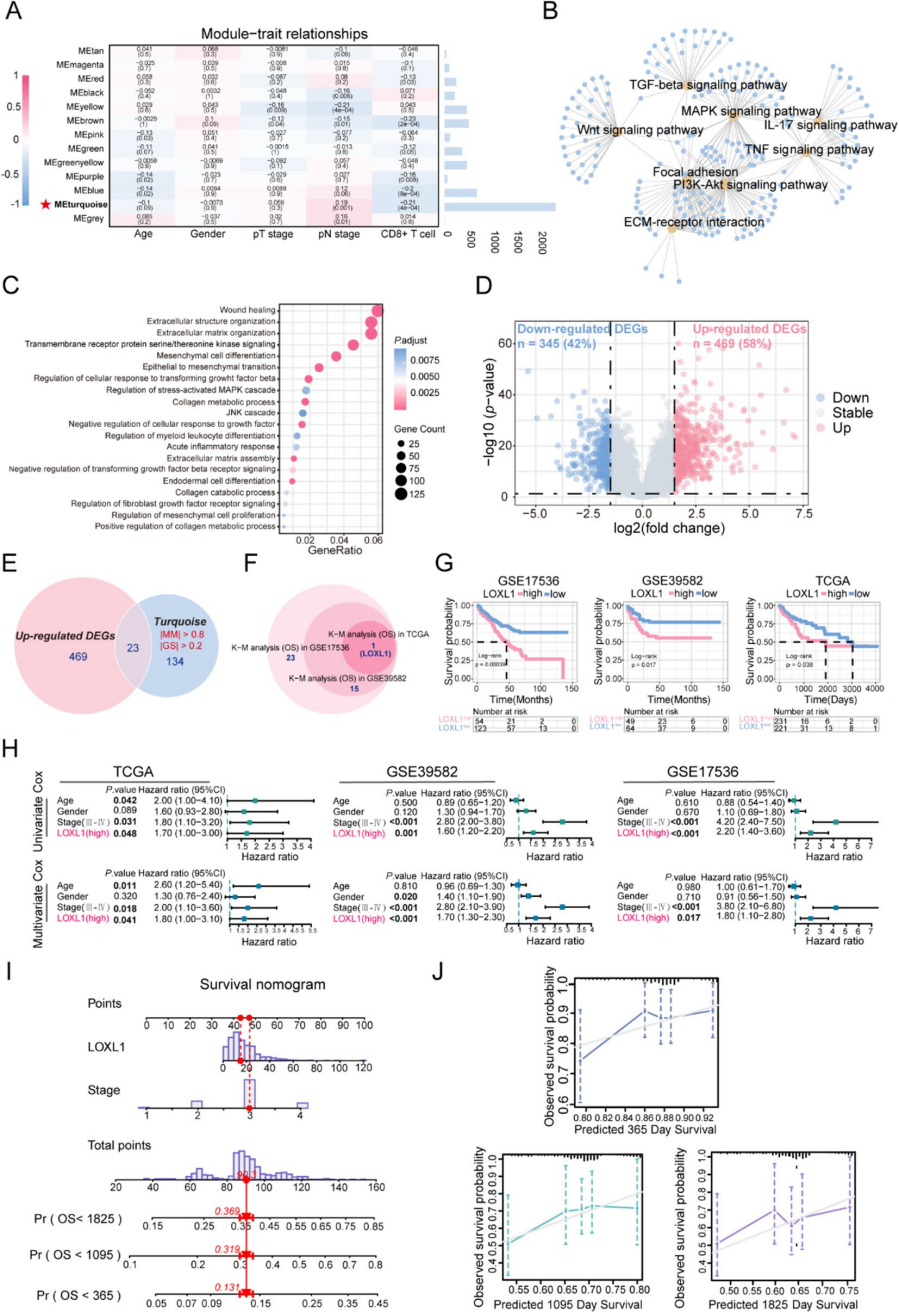
Screening of hub module via weighted gene co-expression network analysis (WGCNA) and identification of LOXL1 as a hub gene. **(A)** Left: Heatmap for the correlation between module eigengenes and clinical traits including pathological T, N stage and CD8 + T cell infiltration level of colorectal cancer (CRC) patients in GSE39582 discovery dataset. Each cell contains corresponding correlation coefficient and *P*-value.* P*-values were calculated using *P*earson’s correlation analysis. The turquoise module was selected as the most significant module which was positively correlated with pN stage and negatively correlated with CD8 + T cell infiltration. Right: The bar chart indicates the number of genes in each module. **(B)** Gene Ontology (GO) biological process (BP) enrichment analysis and **(C)** Kyoto Encyclopedia of Genes and Genomes (KEGG) pathway enrichment analysis for genes in the turquoise module. A *p*-value less than 0.05 indicated statistical significance. **(D)** The GSE39582 validation dataset which contains 19 colorectal adjacent normal tissues and 123 colorectal tumor tissues was used to examine the differentially expressed genes (DEGs) between normal and tumor tissues. 814 DEGs including 469 up-regulated and 345 down-regulated genes were identified and selected to draw a volcano plot. The red and blue dots represent significantly up-regulated and down-regulated genes respectively (|log2FC|> 1.5, *P* < 0.01), and grey dots represent genes without significant expression changes. **(E)** The Venn diagram depicted the overlapping genes between up-regulated DEGs and genes in the turquoise module. A total of 23 overlapping hub genes were obtained. **(F)** The circle plot determined that LOXL1 was the only gene significantly associated with patient’s overall survival in the GSE17536 cohort, GSE39582 cohort and TCGA-COAD cohort. **(G)** The Kaplan–Meier curves showed that high expression of LOXL1 was correlated with poor survival rate of CRC patients in GSE17536 cohort, GSE39582 cohort, and TCGA-COAD cohort. Optimal separation cut-off value was used to achieve best statistical significance. **(H)** Univariate and multivariate Cox proportional hazards regression analysis showed independent factors for overall survival (OS) in TCGA-COAD cohort, GSE39582 cohort and GSE17536 cohort. Forest plot presents the hazard ratio (HR) value and 95% confidence interval (CI). **(I)** A nomogram combining LOXL1 expression and pathological stage was constructed to predict the 1-, 3-, and 5-year overall survival probability of CRC patients. The red line and arrows represent an example of designated points. **(J)** Calibration curves were used to validate the consistency between predicted nomogram results and the actual 1-, 3-, and 5-year survival outcomes. The y-axis represents the measured survival probabilities. The x-axis represents the nomogram-predicted survival probabilities. The diagonal grey solid line represents the ideal nomogram, and the blue, green, purple line represents the 1-, 3-, and 5-year observed nomograms respectively

Since the CD8 + T cell infiltration plays an indispensable role in CRC prognosis, we integrated CD8 + T cell levels (assessed via CIBERSORT) with clinical traits to perform WGCNA. We included a total of 19,028 gene expression profiles of 237 samples from GSE39582 discovery dataset to construct co-expression network after removing outlier samples (Supplementary Fig. 1A). For constructing a scale-free network, we chose a power of β = 4 (yielding a scale-free R2 = 0.9) (Supplementary Fig. 1B). WGCNA clustered all these genes into 12 gene modules using a cluster dendrograms (Supplementary Fig. 1C). Notably, the turquoise module exhibited the highest correlation with both CD8 + T cell infiltration and pN stage in CRC (R = -0.21, P = 4e-04; R = 0.19, P = 0.001, respectively) (Fig. 1A), suggesting genes in the turquoise module may be closely correlated with the malignancy and prognosis of CRC. Scatter plots of MM vs. GS verified the association between turquoise module and CD8 + T cells infiltration or pN stage (Supplementary Fig. 1D-E), leading to its identification as a key module. Subsequent GO and KEGG analyses revealed that the turquoise module genes predominantly participate in wound healing, extracellular matrix organization, epithelial to mesenchymal transition (Fig. 1B, Table S4), and pathways including focal adhesion, TGF-beta signaling, and ECM-receptor interaction, etc. (Fig. 1C, Table S4), indicating the hub module genes might serve critical functions in mediating the TME and metastasis of CRC.

### Identification of LOXL1 as a prognostic hub gene in CRC

To determine the hub genes responsible for CRC prognosis, we firstly examined the GSE39582 cohort and identified 814 DEGs between tumor and para-tumor tissues (Fig. 1D, Table S5), which were found to be involved in mediating signaling like extracellular matrix organization and ECM-receptor interaction (Supplementary Fig. 2A and B, Table S6). Intersection of the 469 up-regulated DEGs with 134 genes from the turquoise module (selected based on MM > 0.8 and GS > 0.2 criteria) led to the identification of 23 candidate genes (Fig. 1E). To assess the prognostic relevance of these 23 genes, KM survival analysis on patients stratified by the expression levels of these 23 genes was implemented in three independent cohorts. The result showed that LOXL1 was the only gene that might affect clinical outcome (Fig. 1F, Table S7). The KM curve in the three cohorts demonstrated that high LOXL1 expression was significantly linked with r worse OS (Fig. 1G). To further confirm the prognosis prediction performance of LOXL1, its association with OS in CRC patients was evaluated using univariate and multivariate Cox regression analysis. The analyses confirmed that high LOXL1 expression, along with advanced pathological stage, were significant risk factors associated with poor prognosis. Notably, LOXL1 expression remained an independent prognostic factor even after adjustment for other clinical risk factors (Fig. 1H). Additionally, with a goal of predicting the survival probability for CRC patients in clinic, a risk estimation nomogram based on the above two independent risk factors (tumor stage and LOXL1) was established and scores were calculated to predict 1-, 3- and 5-year OS for individual patient (Fig. 1I). Calibration plots revealed a high level of concordance between predictive and observed outcomes, indicating the good performance of the nomogram in predicting patient’s OS (Fig. 1J).

### LOXL1 expression was positively correlated with TNM staging and poor differentiation

**Fig. 2:**
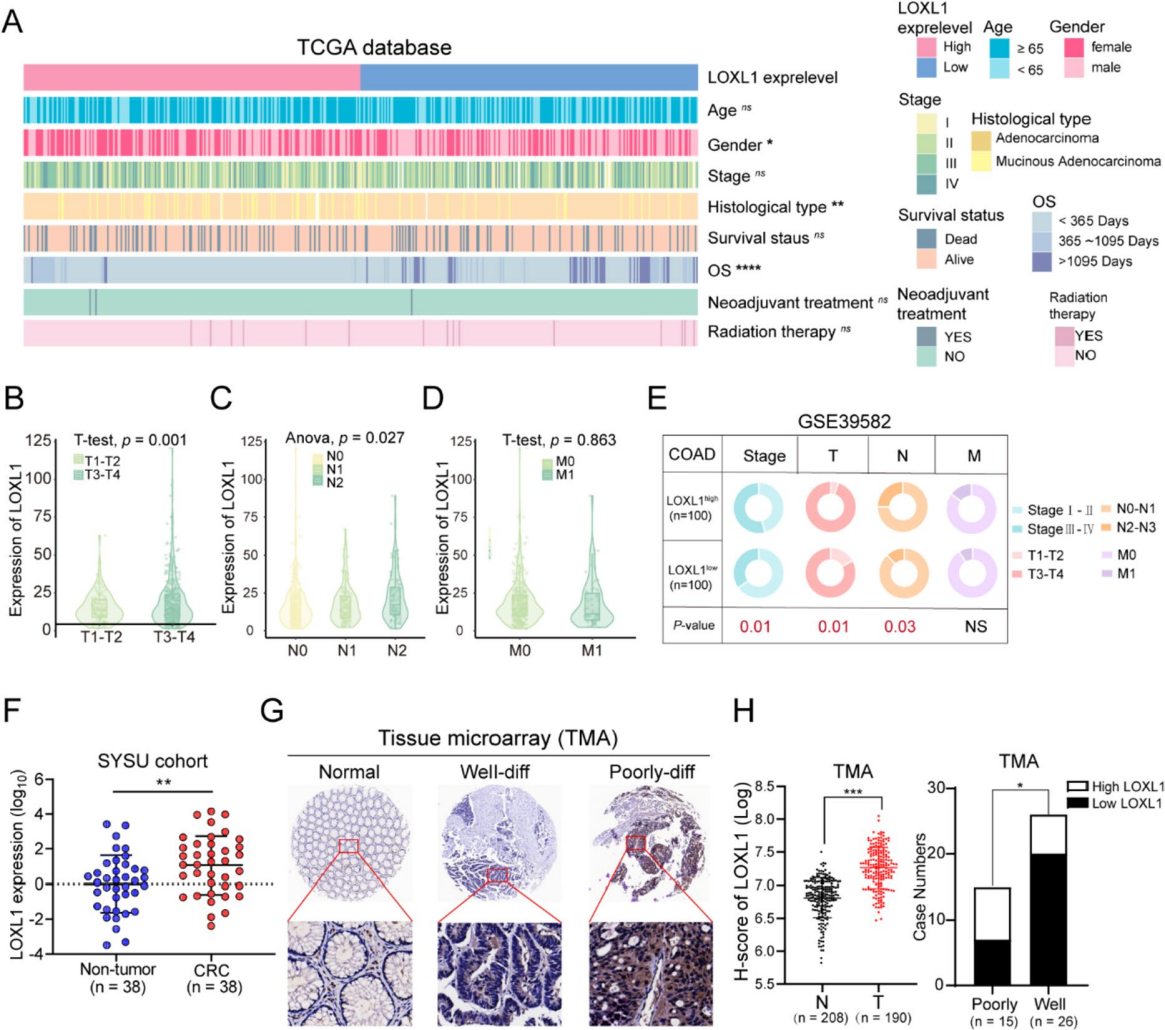
The correlation between LOXL1 expression and clinicopathological characteristics of CRC patients. **(A)** Heatmap visualizing the distribution of clinicopathological features in patients divided by the expression level of LOXL1 from TCGA-COAD cohort. White lines represent missing values. **(B-D)** The LOXL1 expression level in TCGA CRC patients stratified by pathological T, N, and M stage. **(E)** The circular pie chart shows the proportion difference of clinical indices between LOXL1 high and low expression groups from the GSE39582 cohort. Chi-squared test was used for statistical analysis, ns: not significant, ^*^P < 0.05, ^**^P < 0.01, ^***^P < .001. **(F)** Relative mRNA expression of LOXL1 was quantified in a cohort of surgically resected human CRC tissues and paired non-tumor tissues (n = 38) through quantitative real-time PCR, ^**^P < 0.01. Statistical significance was determined by the paired student’s t test and error bars represent standard deviations. **(G)** IHC staining for LOXL1 expression was performed on tissue microarray (TMA) containing normal colon tissues, well differentiated- and poorly differentiated CRC tissues from the SYSU cohort. **(H)** (left) Quantification of LOXL1 expression in normal or tumor tissues based on IHC results of the TMA. H-score represents the immunostaining score obtained by Image Pro Plus software. Statistical significance was assessed using the unpaired student’s t test. (right) High expression of LOXL1 significantly associated with poor differentiation status (Chi-squared test, ^*^P < 0.05). “Poorly” represents poor and moderate-poor differentiation status, and “Well” represents well and well-moderate differentiation status

The role of LOXL1 in CRC malignancy was evaluated. In TCGA-COAD cohort, the distribution of clinicopathological features including age, gender, stage, histological type, survival status, OS time, neoadjuvant treatment, and radiation therapy were examined in patient groups with high or low LOXL1 expression. The results suggested that elevated LOXL1 expression was inversely associated with OS duration (Fig. 2A, Table S8), and markedly higher in patients exhibiting advanced pT and pN stage, while no significant difference was observed for the pM stage (Fig. 2B-D). Similar results were obtained when analyzing the GSE39582 cohort. Larger tumor size (T3-4), regional lymph nodes metastasis (N2-3) and advanced tumor stage (stage III-IV) were more commonly detected in patients with high LOXL1 expression (Fig. 2E). Furthermore, quantitative RT-PCR analysis of 38 clinical sample pairs revealed a pronounced upregulation of LOXL1 mRNA in CRC tissues, compared with non-tumor tissues (Fig. 2F). IHC assessments on a tissue microarray containing 208 pairs of CRC tissues corroborated the augmented LOXL1 protein expression in tumor tissues, with a significant association to poorer differentiation status (Fig. 2G and H).

### LOXL1 was involved in mediating epithelial-mesenchymal transition in CRC

**Fig. 3:**
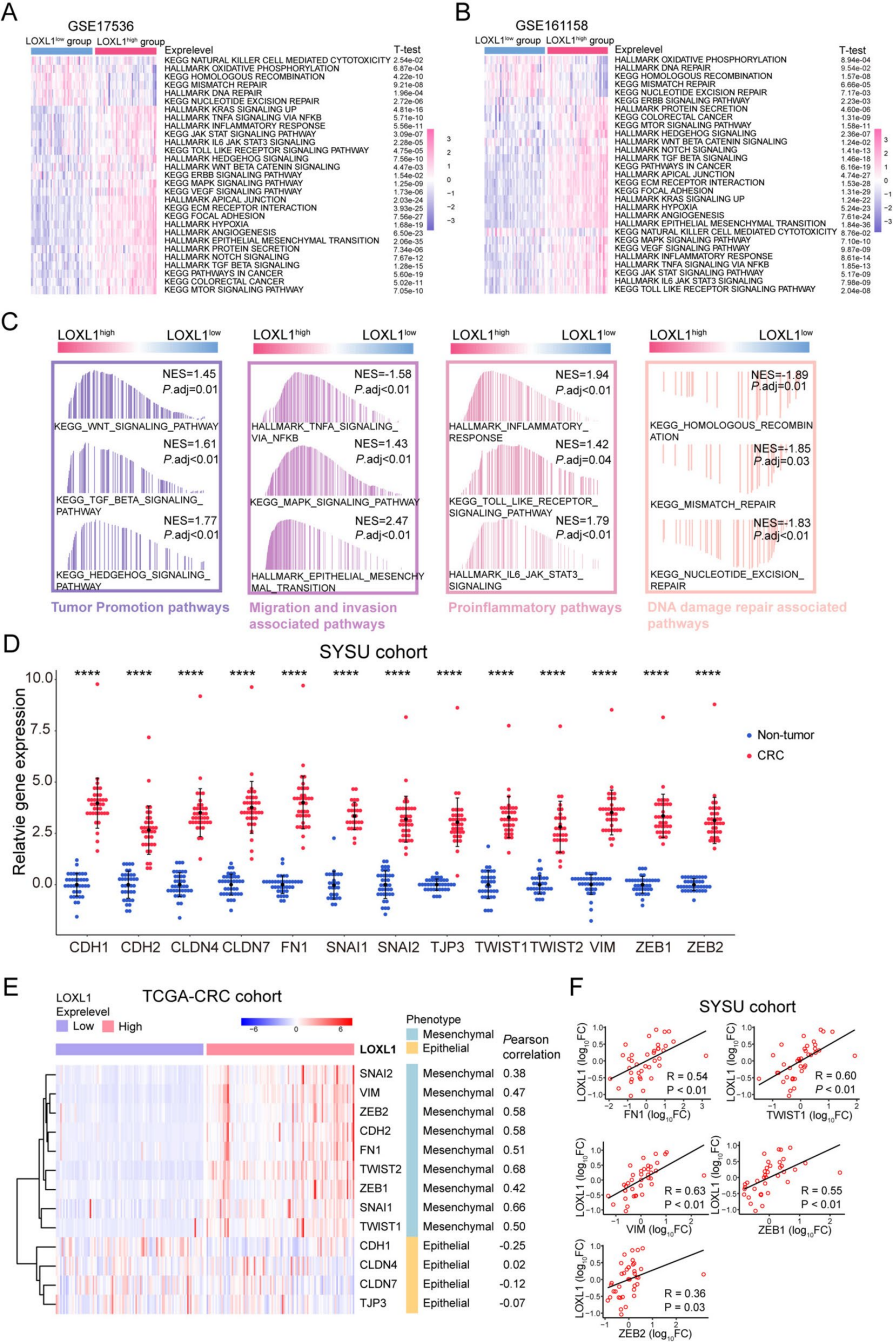
Biological pathway enrichment analysis of LOXL1 and its correlation with epithelial-mesenchymal transition (EMT) in CRC. **(A-B)** Gene set variation analysis (GSVA) revealed the activation or inhibition status of HALLMARK terms and KEGG pathways in LOXL1-high and LOXL1-low groups from GSE17536 (**A**) and GSE161158 (**B**) cohorts. Samples were categorized according to the median expression level of LOXL1 gene. The statistical significance of differences was determined by Student’s *t* test. **(C)** Gene set enrichment analysis** (**GSEA) revealed the activation or inhibition of signaling pathways in LOXL1-high or LOXL1-low groups from the TCGA-COAD database. **(D)** Expression levels of EMT associated genes in non-tumor and CRC samples from the SYSU cohort by qRT-PCR, ^****^P < .0001. The Student’s t test was used for statistical analysis. **(E)** Heatmap depicting the differential expression pattern of epithelial or mesenchymal markers in LOXL1-high and LOXL1-low groups from the TCGA-COAD cohort. *P*earson correlation coefficient of gene expression between LOXL1 and epithelial or mesenchymal markers are shown on the right. **(F)** Scatter plots demonstrating the positive correlation between LOXL1 and EMT related genes (FN1, TWIST1, VIM, ZEB1, ZEB2) from the SYSU cohort by qRT-PCR. *P*-values were determined using *P*earson correlation analysis

To investigate the association between LOXL1 expression and tumor biological functions, GSVA was performed in two independent cohorts. Results demonstrated that carcinogenic activation-related pathways, such as epithelial-mesenchymal transition (EMT), focal adhesion, ECM receptor interaction, angiogenesis, and TGF-β signaling were predominantly activated in the LOXL1 high expression group, while the NK cell mediated cytotoxicity was overrepresented in LOXL1 low group (Fig. 3A and B), indicating the involvement of LOXL1 in mediating immune cell function and tumorigenesis.

Then we performed GSEA analysis in TCGA-COAD dataset. The top 21 enriched pathways in either LOXL1 high or low expression group were obtained (Table S9). As expected, programs associated with tumor promotion, migration and invasion, as well as proinflammatory responses were prominently enriched in the high LOXL1 expression group, while DNA damage repair associated pathways were upregulated in LOXL1 low expression group (Fig. 3C), suggesting the oncogenic function of LOXL1 in CRC. Given the known involvement of lysyl oxidase (LOX) family members in EMT, the relationship between LOXL1 expression and various EMT markers was examined. A variety of markers were selected based on their relatively high expression in CRC tissues (Fig. 3D). Results demonstrated that LOXL1 correlated positively with various mesenchymal markers and negatively with epithelial markers in TCGA-CRC database (Fig. 3E). Furthermore, qRT-PCR was performed in our own cohort to validate the result. Moderate/strong correlations were observed between LOXL1 and FN1, TWIST1, VIM, ZEB1, and ZEB2 in 38 CRC tissues (Fig. 3F).

### LOXL1 expression was significantly associated with TMB and genomic instability

Since genomic instability and TMB are hallmarks of malignancy (Hanahan and Weinberg 2011), we examined the impact of TMB on CRC prognosis and found no marked difference in OS when comparing high versus low TMB groups (Supplementary Fig. 3A). Intriguingly, elevated LOXL1 expression corresponded with increased TMB, as evidenced by our findings (Supplementary Fig. 3B and C). This link was statistically significant, with a Spearman coefficient R of 0.11 and a P-value of 0.026 (Supplementary Fig. 3D). The distribution of CRC somatic variants in LOXL1-high or low-expression patient groups were profiled. Missense mutations were the most common mutation type (Supplementary Fig. 3E), and C > T occurred most frequently among all single-nucleotide variants (Supplementary Fig. 3F). In addition, an analysis of the top 20 driver genes with the highest mutation frequencies revealed a pattern of co-occurrence (Supplementary Fig. 3G). Not surprisingly, patients with increased LOXL1 expression exhibited a higher overall mutation frequency compared to those with lower expression (Supplementary Fig. 3H and I, Table S10).

### LOXL1 promoted CRC proliferation, migration and invasion

**Fig. 4:**
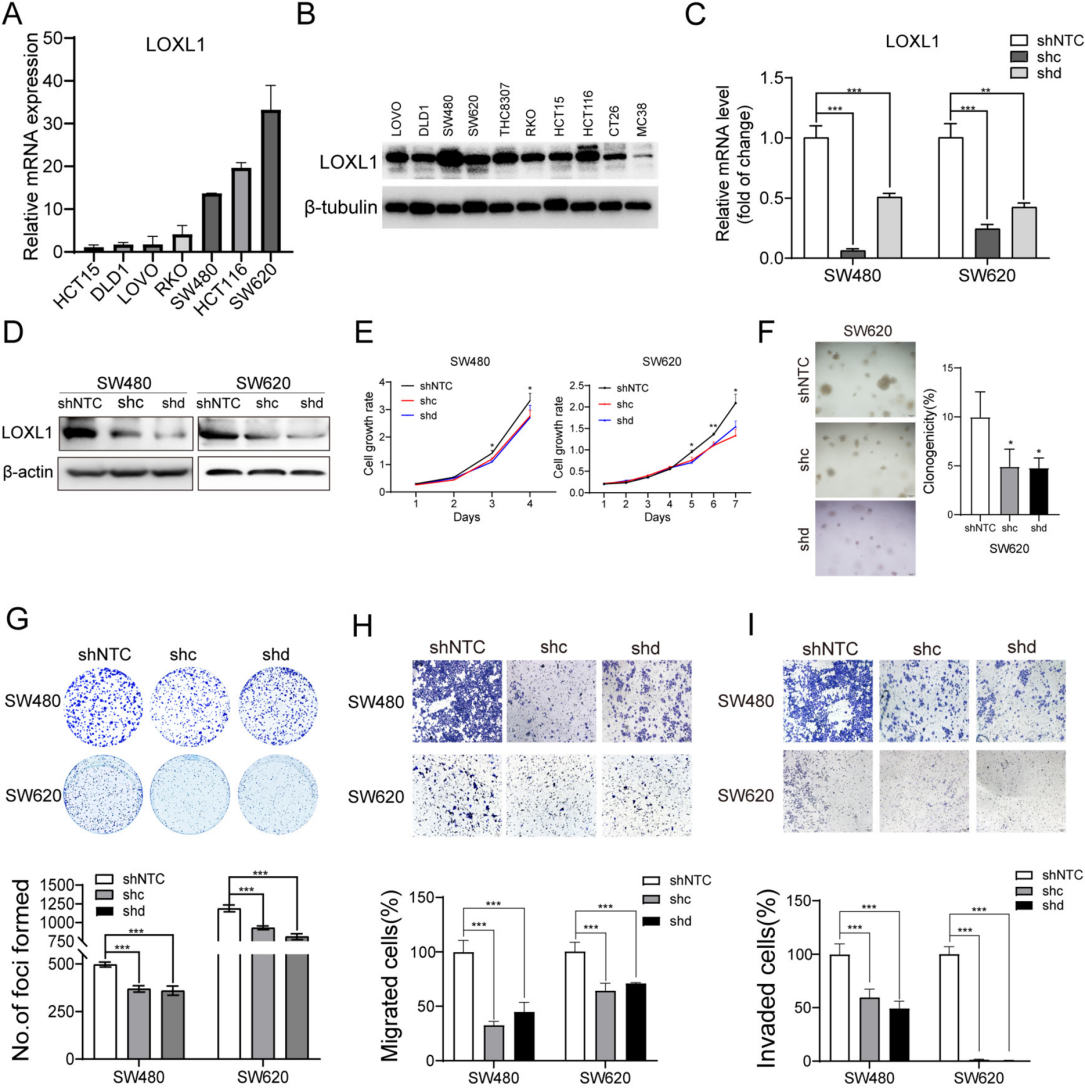
Loss of LOXL1 reduces proliferation, migration and invasion of CRC cells in vitro. **(A-B)** Relative mRNA **(A)** and protein **(B)** levels of LOXL1 in various colorectal cancer cell lines were quantified. 18S was used as a loading control for qRT-PCR, and β-tubulin was used as a loading control for Western blot. **(C-D)** Silencing efficiency of two shRNAs targeting LOXL1 (shc, shd) in SW480 and SW620 cells was assessed using qRT-PCR **(C)** and western blot **(D)**, with β-actin serving as the loading control for Western blot. **(E)** The effect of LOXL1 silencing on SW480 and SW620 cells proliferation were evaluated by the CCK-8 assay. Statistical significance: ^*^P < 0.05, ^**^P < 0.01. **(F)** Representative images of the soft agar colony formation assay in SW480 and SW620 cells after transfection with shNTC or shLOXL1 (left). Quantification analysis of clonogenicity was depicted in the bar chart (right). Statistical significance: ^*^P < 0.05. **(G-I)** SW480 and SW620 cells transfected with shNTC or shc/shd targeting LOXL1 were subjected to foci colony formation **(G)**, migration **(H)**, and invasion assays **(I)**. Quantification analysis of experimental results are presented in the lower panel. The unpaired students’ t test was used for statistical analysis, ^***^P < .001

Our investigation into LOXL1's oncogenic role in CRC involved loss-of-function studies. We initially assessed LOXL1's mRNA and protein levels across various CRC cell lines using qRT-PCR and Western blot analyses (Fig. 4A and B). CRC cell lines with relatively high LOXL1 expression (SW480 and SW620) were selected for loss-of-function studies. The silencing effect of LOXL1 by two shRNAs was determined (Fig. 4C and D). Compared with control cells (shNTC-transfected cells), knockdown of LOXL1 (both shc- and shd- transfected cells) significantly impaired proliferation, colony formation in soft agar, foci formation, migratory and invasive capabilities of CRC cells (Fig. 4E-I). These findings collectively underscore LOXL1's role in promoting CRC proliferation and its metastatic properties.

### Elevated LOXL1 expression correlate with diminished CD8 + T Cell infiltration

**Fig. 5:**
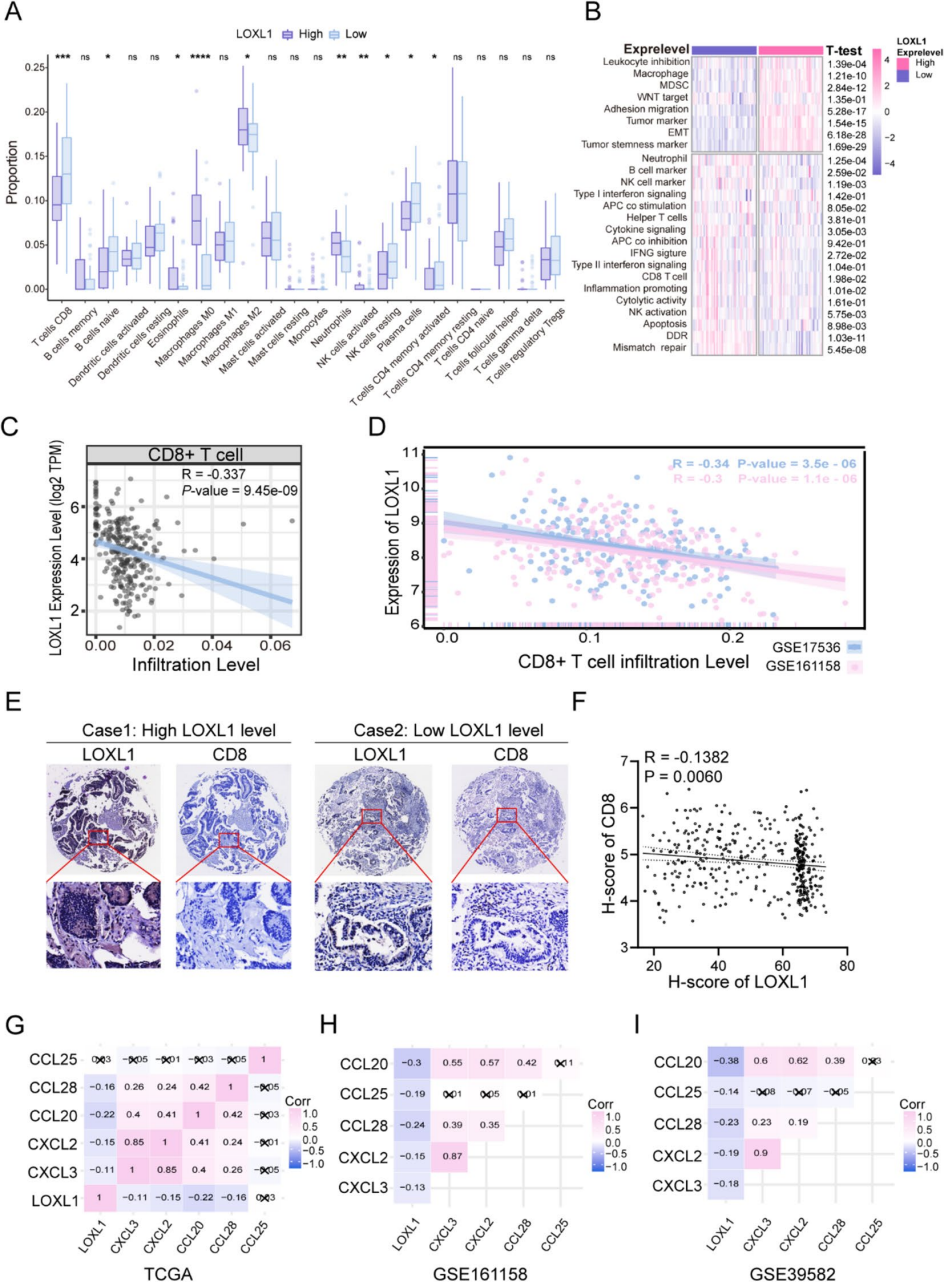
The distinct landscape of tumor immune microenvironment between patients with high or low LOXL1 expression. **(A)** Boxplot of 22 immune cell abundance based on deconvolution by CIBERSORT between high and low LOXL1 expression groups in the GSE17536 dataset. Samples were categorized according to the median expression level of LOXL1 gene. The upper and lower ends of the boxes are the 25th and 75th percentiles (interquartile range) respectively. The lines within the boxes represents the median value, and the scattered dots represent outliers. The significance of differences was determined by the Wilcoxon test (ns: not significant, *P < 0.05, ** *P* < 0.01, *** *P* < 0.001, **** *P* < 0.0001). **(B)** The heatmap showed the enrichment level of 25 immune-related gene sets based on single sample gene set enrichment analysis (ssGSEA) analysis in the high and low LOXL1 expression groups. The Student’s *t* test was used for statistical analysis. **(C-D)** LOXL1 expression was negatively correlated with the infiltration level of CD8 + T cells in TCGA-COAD cohort (Cor = -0.337, *P* = 9.45e-09) **(C)**, GSE17536 cohort (Cor = -0.34, *P* = 3.5e-06), and GSE161158 cohort (Cor = -0.3, *P* = 1.1e-06) **(D)**. **(E)** IHC staining of LOXL1 and CD8 in TMA comprising 208 pairs of CRC tissues. Representative IHC staining images from two cases were displayed. **(F)** Correlation analysis of protein expression between LOXL1 and CD8 in CRC patients using *P*earson correlation analysis. H-score represents the value of IOD sum or IOD/Area determined by image pro plus software. **(G-I)** The heatmap revealed the expression correlation between LOXL1 and multiple chemokines in TCGA-COAD cohort **(G)**, GSE161158 cohort **(H)**, and GSE39582 cohort **(I)**. The *P*earson’s correlation coefficient was calculated and demonstrated in each cell, and cells with *P* value ≥ 0.05 were marked with cross

Comprehensive annotation of biological processes and signaling pathways suggested the role for LOXL1 in modulating the tumor immune microenvironment in CRC. Utilizing the CIBERSORT algorithm, we explored the relationship between LOXL1 expression and immune cell infiltration. It was observed that adaptive immune cell, notably CD8 + T cells known for their positive impact on survival and immunotherapy response in various cancers (Bruni et al. 2020), was markedly lower in cases with heightened LOXL1 expression (Fig. 5A). Manually curated immune associated signatures representing diverse immune cell types and molecular functions were analyzed by ssGSEA algorithm. LOXL1 high expression group was characterized by escalated infiltration of immunosuppressive components, while LOXL1 low expression group was linked to a richer infiltration of anti-tumor immune cell signatures, including CD8 + T cells and interferon-γ signatures (Fig. 5B). Furthermore, LOXL1 expression was found to be inversely correlated with CD8 + T cells infiltration in several independent cohorts (Fig. 5C and D).

The protein expression level of LOXL1 and CD8 + T cell infiltration were also investigated by IHC staining in tissue microarray comprising 208 pairs of CRC tissues. Consistently, we found that there existed a negative correlation between LOXL1 expression and CD8 + T cell infiltration (Fig. 5E and F). Since chemokines might facilitate the recruitment of diverse immune cells into tumors (Turner et al. 2014), we sought to determine whether there exists a correlation between LOXL1 expression and chemokines expression. The results revealed that CXCL2, CXCL3, CCL20, CCL25 and CCL28, which played key role in recruiting T lymphocytes to tumor microenvironment (Tosti et al. 2020; Chen, et al. 2020; Gong et al. 2019), were negatively correlated with LOXL1 expression in in dependent cohorts (Fig. 5G-I). Taken together, LOXL1 might be involved in mediating the immuno-suppressive microenvironment leading to poor prognosis of CRC patients.

### High expression of LOXL1 predicts poor clinical outcomes of ICB

**Fig. 6:**
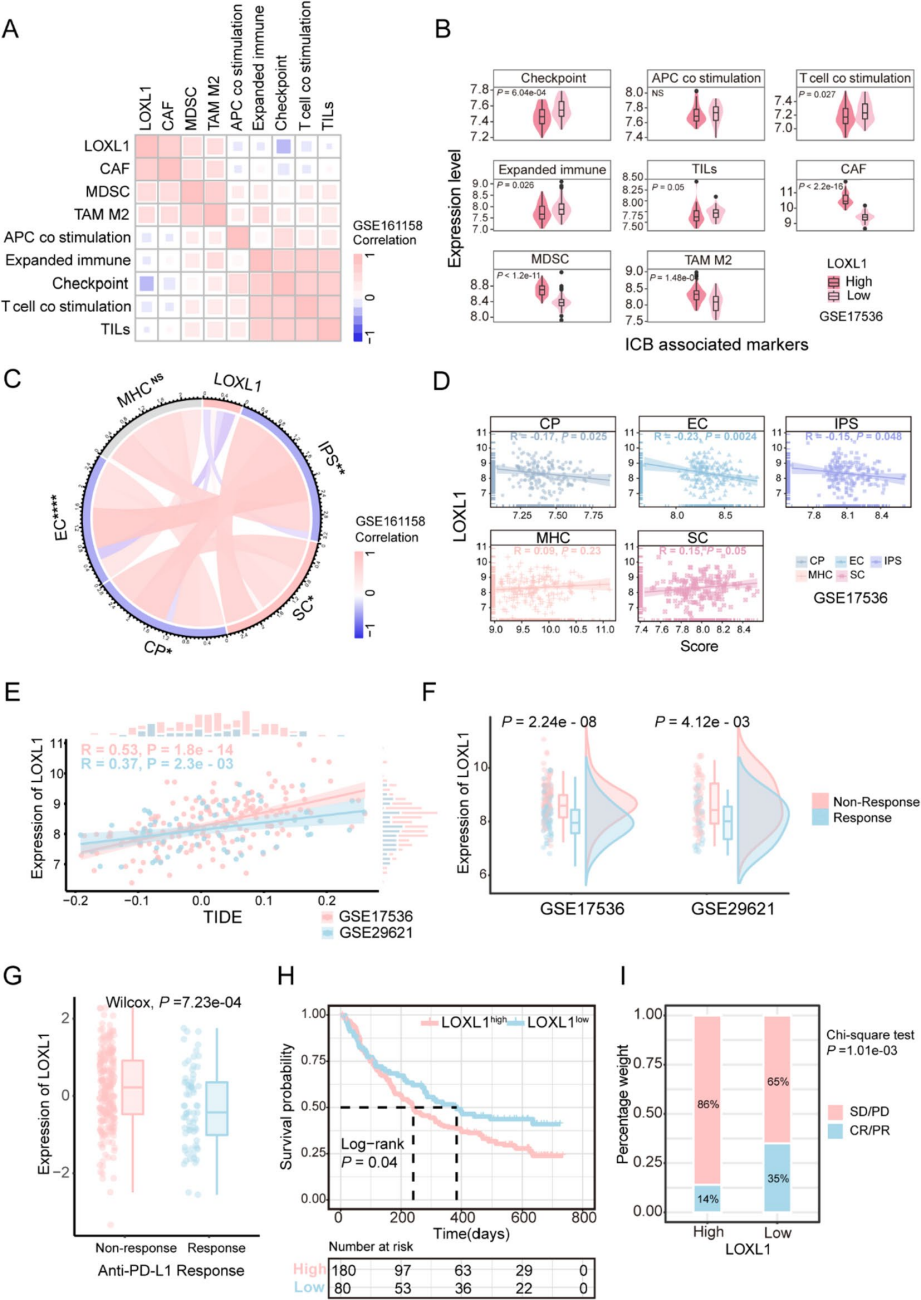
The expression of LOXL1 could predict patients’ responsiveness to immune checkpoint blockade (ICB) treatment and prognosis. **(A)** *P*earson correlation analysis was used to determine the correlation between LOXL1 expression and key immune modulators in GSE161158 cohort. The square color indicates correlation coefficients, and the square size represents the statistical *P* value, with larger size indicating greater statistical significance. **(B)** Violin plots comparing the expression levels of key immune modulators between the high and low LOXL1 expression tumors in GSE17536 cohort. The statistical analysis was determined by Wilcoxon test. **(C)** Chord diagram illustrated the association between LOXL1 expression and MHC molecular (MHC), effector cell (EC), immunosuppressive cell (SC), immunophenoscore (IPS) score in GSE161158 cohort. Colors indicate correlation coefficients. *P*earson correlation analysis was used for statistical analysis (ns: not significant, *P < 0.05; **P < 0.01; ***P < 0.001, **** *P* < 0.0001). **(D)** The correlation between LOXL1 expression and MHC, EC, SC, IPS score in GSE17536 cohort. **(E)** LOXL1 expression was positively associated with tumor immune dysfunction and exclusion (TIDE) scores representing ICB responsiveness in GSE17536 cohort and GSE29621 cohort. **(F)** Differences in LOXL1 expression between putative immunotherapeutic responders and non-responders from TIDE in GSE17536 cohort and GSE29621 cohort. **(G)** Comparison of LOXL1 expression between patients responding or not responding to anti-PD-L1 blockade immunotherapy in IMvigor210 cohort. Wilcoxon test was used for statistical analysis. **(H)** Kaplan–Meier curve showed that high LOXL1 expression predicted worse survival outcome in IMvigor210 cohort (log-rank test, *P* = 0.04). **(I)** Stacked bar graph indicated that LOXL1 expression was significantly associated with poor treatment response to anti-PD-L1 immunotherapy in IMvigor210 cohort. (chi-square test,* P*-value = 1.13E-3). CR, complete response; PR, partial response; SD, stable disease; PD, progressive disease

Considering the immunosuppressive role of LOXL1 in CRC, we wondered whether LOXL1 could predict the clinical efficacy of immunotherapy. The association between LOXL1 levels and previously published gene signatures associated with ICB response (Auslander et al. 2018) were examined in GSE161158 and GSE17536 cohorts. The result revealed a negative correlation between LOXL1 expression and favorable gene signatures associated with ICB response, such as tumor-infiltrating lymphocytes (TILs). Conversely, a positive correlation with the presence of immune-suppressive myeloid-derived suppressor cells which might suppress immune response was observed (Fig. 6A and B). The IPS have been reported to potently predict patients’ response to ICB based on immunogenicity (Charoentong et al. 2017). We then explore the correlation between LOXL1 expression and IPS in CRC patients. We found that as LOXL1 expression elevated, EC (effector cells) score, CP (immune checkpoint) score, and IPS (immunophenoscore) declined, while the SC (suppressor cells) score increased in two independent cohorts (Fig. 6C and D). The TIDE approach was also utilized and revealed a significant positive correlation between the levels of LOXL1 expression and the TIDE scores (Fig. 6E). In addition, LOXL1 expression was found to be substantially elevated in the group of patients who did not respond to treatment compared to those who did, as indicated by the TIDE analysis (Wilcoxon test, P < 0.001) (Fig. 6F), suggesting the potential of LOXL1 as a predictive biomarker for the effectiveness of ICB therapy.

To verify whether LOXL1 could be used as a biomarker, an anti-PD-L1 immunotherapy cohort (IMvigor210) with detailed clinical data was adopted. Interestingly, a lower LOXL1 expression level was associated with enhanced responses to anti-PD-L1 therapy, in contrast to those with higher LOXL1 levels who exhibited negligible response (Wilcoxon test, *P* = 7.23e-04) (Fig. 6G). A Kaplan–Meier survival analysis further established a significant inverse relationship between LOXL1 expression and survival outcomes following anti-PD-L1 treatment (Log- rank test, *P* < 0.05) (Fig. 6H). Moreover, our analysis revealed a markedly higher occurrence of complete or partial response (CR/PR) in patients with lower LOXL1 expression (35%) compared to those with higher expression (14%), as confirmed by a Chi-square test (*P* = 1.01e-03) (Fig. 6I). Collectively, these results suggested the predictive role for LOXL1 in clinical benefit of ICB.

### Overview of LOXL1 in pan-cancer

**Fig. 7:**
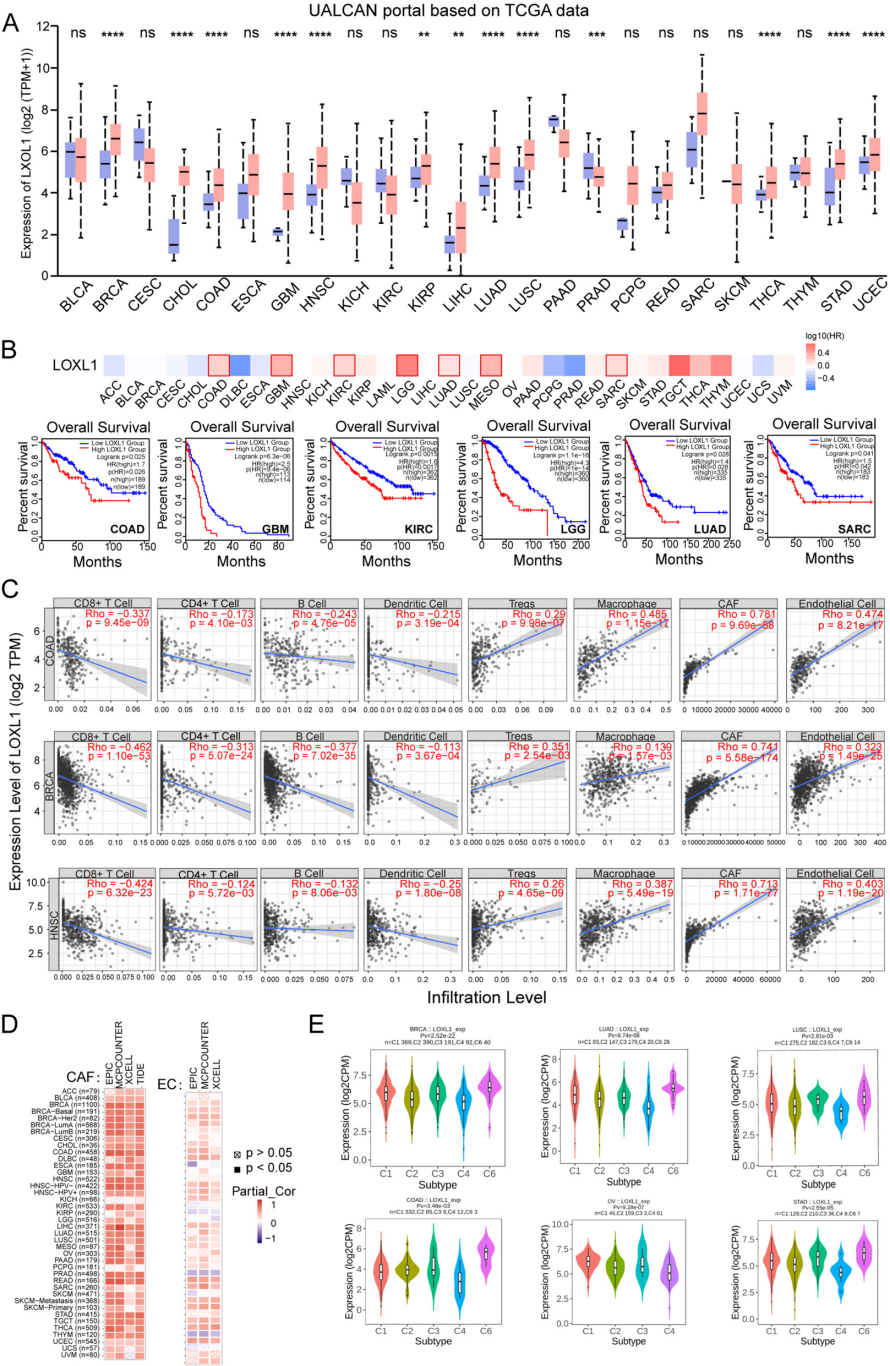
Role of LOXL1 in pan-cancer. **(A)** The expression levels of LOXL1 in tumor tissues and corresponding normal tissues from UALCAN database. Student’s *t* test was used for statistical analysis (ns: not significant, *P < 0.05, **P < 0.01, ***P < 0.001 and ****P < 0.0001). **(B)** The upper panel showed the survival map using the online tool of Gene Expression Profiling Interactive Analysis (GEPIA2). The Kaplan–Meier survival plots in the lower panel indicated that high LOXL1 expression correlated with poor survival outcome in different kinds of cancer (COAD, GBM, KIRC, LGG, LUAD and SARC). **(C)** Correlation of LOXL1 expression with the infiltration level of immune cells in COAD, BRCA and HNSC. **(D)** *P*earson correlation was analyzed between LOXL1 expression and the infiltration of cancer-associated fibroblast (CAF) (left panel) and endothelial cell (EC) (right panel) based on the EPIC, MCPCOUNTEER, XCELL and TIDE algorithms. **(E)** Comparison of LOXL1 expression among different immune infiltration subtypes in multiple cancers from the Tumor–Immune System Interactions and Drug Bank (TISIDB) database. (C1, wound healing; C2, IFN-gamma dominant; C3, inflammatory; C4, lymphocyte depleted; C5, immunologically quiet; and C6, TGF-b dominant)

To examine the prognostic value and immune regulatory influence of LOXL1 across pan-cancer, we analyzed its expression in 24 solid tumor types from TCGA database. The results, which is sourced from UALCAN, revealed that LOXL1 was dysregulated in 13 types of cancers, with 12 of them showing significant upregulation (Fig. 7A). Analysis of data from multiple databases, including UALCAN, TIMER 2.0, and TNMplot, consistently indicates an upregulation of LOXL1 expression in colorectal cancer compared to normal tissue (Supplementary Fig. 4A-D). Metastatic tumors displayed elevated expression of LOXL1 compared with primary tumors and normal tissues across several cancers from TNM plotter database (Supplementary Fig. 5A). Additionally, LOXL1 expression was found increased with tumor progression from early stage to advanced stage (Supplementary Fig. 5B). Kaplan–Meier analysis was then performed and the results demonstrated that high LOXL1 expression predicted shorter OS and DFS in a variety of cancer types (Fig. 7B, Supplementary Fig. 5C). The genetic alterations of LOXL1 in pan-cancer were also assessed using cBioPortal database. The most common alteration type of LOXL1 was “amplification” in the majority of tumors, and multiple types of LOXL1 mutations were observed (Supplementary Fig. 5D and E).

Additionally, LOXL1 expression and immune cell infiltration were correlated using TIMER2.0 database. Negative correlation was found between LOXL1 expression and the infiltration abundance of anti-tumor immune cells such as CD8 + T cell, CD4 + T cell, B cell, and dendritic cell in COAD, BRCA, and HNSC. Conversely, LOXL1 expression positively correlated with a series of immunosuppressive cells including Tregs, and macrophage (Fig. 7C). Remarkably, cancer-associated fibroblast and endothelial cells, which have been reported to promote tumor progression (Chen and Song 2019; Yang et al. 2021), exhibited strong positive correlations with LOXL1 expression in the majority of cancers (Fig. 7D), indicating the immune-regulatory role of LOXL1 in cancers. Additionally, using the TISIDB database, we explored LOXL1 expression across different immune subtypes in various cancers. Tumors were categorized into six immune subtypes: C1 (wound healing), C2 (IFN-gamma dominant), C3 (inflammatory), C4 (lymphocyte depleted), C5 (immunologically quiet), and C6 (TGF-β dominant) (Thorsson et al. 2018). Our findings revealed a marked enrichment of LOXL1 in wound healing and TGF-β dominant subtypes and a downregulated in IFN-γ dominant subtypes in cancers including colorectal (Fig. 7E), suggesting LOXL1’s immunosuppressive role in pan-cancer.

## Discussion

Immunotherapy, particularly promising for metastatic colorectal cancer (CRC) with mismatch repair deficiencies and high microsatellite instability, requires effective immune infiltration for success. This underscores the clinical imperative to identify prognostic biomarkers differentiating highly immunogenic "hot tumors" from less responsive "cold ones." In the current study, LOXL1 was identified as a novel potential predictor for the prognosis and immunotherapy response of CRC patients using WGCNA based on public transcriptomic datasets. We comprehensively unveiled the correlation between LOXL1 expression and prognosis, clinicopathological features, tumor molecular characteristics, and tumor immune microenvironment in CRC patients. Notably, this is the first study to report the potential role of LOXL1 in remodeling the TME and predicting immunotherapy efficacy in CRC.

Previous studies have revealed that LOXL1 dysregulation was correlated with advanced-stage cancer and worse prognosis, suggesting a link between LOXL1 expression and tumor progression in various cancers (Ye et al. 2020). By analyzing CRC samples from the TCGA cohort, we observed prominent enrichment in oncogenesis and metastasis-related pathways across all LOXL1 high expression samples, including EMT process, IL-6/JAK/STAT3 signaling, Hedgehog pathway, etc. Consistently, a wealth of data suggested that aberrant hyperactivation of the IL-6/JAK/STAT3 pathway could drive the metastasis of CRC cells while severely hindering the antitumor immune response in the TME (Wang and Sun 2014). Besides, Hedgehog pathway activation seems to be a common event in CRC which exert a crucial impact on tumor initiation and metastatic cascade (Mazumdar et al. 2011). Therefore, aberrant activations of the above signaling pathways in the LOXL1 high expression groups are responsible for aggressive pathological features and poor prognosis of CRC patients. Further, it was reported that LOXL1 could regulate the ECM remodeling and tumor metastasis by interacting with several ECM proteins like BMP-1 and fibronectin (Grau-Bové et al. 2015). LOXL1 could also stabilize the molecular chaperone regulator BAG2 and impede the apoptosis of cancer cells (Yu et al. 2020). Interestingly, increased production of LOXL1 from CAFs regulated by tumor cell-derived TGF-β and SMAD signaling pathway activation could enhance tumor metastasis via ECM remodeling (Zeltz, et al. 2019). However, the elaborate mechanisms of how LOXL1 mediates tumor metastasis and progression in CRC are still enigmatic and need to be studied in-depth.

Given the importance of the interaction between tumor and TME in tumor progression and response to immunotherapy, the role of LOXL1 in anti-tumor immunity was also systematically evaluated. We observed distinct tumor-associated immune cells compositions in CRC samples with varying LOXL1 expression levels. Specifically, M2 macrophages, M0 macrophages, neutrophil, and eosinophils were more abundant in LOXL1 high expression group, while CD8 + T cell, CD4 + memory activated T cells, B cells, and plasma cells were more common in LOXL1 low expression group. Besides, myeloid-derived suppressor cells (MDSC) were also enriched in LOXL1 high expression group compared to LOXL1 low group, which usually expand during tumorigenesis and exert immunoinhibitory activity by suppressing anti-tumor CD8 + T cells (Jiang et al. 2020; Gabrilovich et al. 2012; Cui et al. 2013). There is still a long way to go on how LOXL1 regulates and reshapes the TME landscape. Previous study revealed that LOXL1 could be upregulated by TGF-β secreted by tumor cells(Lu et al. 2010). Given that TGF-β is an immunosuppressive cytokine that regulates various immune cell types, including promoting the expansion of Treg cells, inhibiting natural killer (NK) cells, and regulating the functions of macrophages(Batlle and Massague 2019), it is highly plausible that LOXL1 plays a significant role in TGF-β-mediated immunosuppression. Our study revealed that LOXL1 expression was associated with the expression of several chemokines like CCL20 and CCL28. The CCL20-CCR6 axis has long been known to be involved in cancer progression by remodeling the TME through regulating immune cell infiltration (Kadomoto et al. 2020). Hence, we presumed that LOXL1 might be a critical contributor to the regulation of chemokine secretion and immune infiltration.

While a comprehensive analysis on the role of LOXL1 in the progression and immunoregulation in CRC has been performed, there are still some limitations in this study. First, the data we used were acquired from the public retrospective databases and more in vivo functional experiments should be conducted to validate the effects of LOXL1 on cancer progression and anti-tumor immunity in the future study. Second, it is also pivotal to explore the underlying molecular mechanisms by which LOXL1 modulates tumor immune microenvironment and consequently affects immunotherapy efficacies. Third, to better verify the predictive value of LOXL1 in responses to immunotherapy, a large scale of prospective cohorts should be enrolled to confirm our findings.

In conclusion, our study found that LOXL1 is a reliable biomarker which could predict prognosis and response to ICB therapy in CRC patients. We characterized not only the genetic alterations of LOXL1 but also its role in remodulating the TME, which might help to boost the efficacy of immunotherapy and develop new targeted therapeutic strategies in CRC.

## Supplementary Information

Below is the link to the electronic supplementary material.

Supplementary file1 (DOCX 17 KB)

Supplementary file2 (XLSX 21 KB)

Supplementary file3 (XLSX 10 KB)

Supplementary file4 (XLSX 14 KB)

Supplementary file5 (XLSX 15 KB)

Supplementary file6 (XLSX 43 KB)

Supplementary file7 (XLSX 13 KB)

Supplementary file8 (XLSX 10 KB)

Supplementary file9 (DOCX 14 KB)

Supplementary file10 (XLSX 15 KB)

Supplementary file11 (XLSX 10 KB)

## Data Availability

The datasets used and/or analyzed during the current study are available from the corresponding author on reasonable request.

## Abbreviations

*ANOVA*: One-way analysis of variance
*BRCA*: Breast invasive carcinoma
*COAD*: Colon adenocarcinoma
*CP*: Immune checkpoint
*CR*: Complete response
*CRC*: Colorectal cancer
*DEGs*: Differentially expressed genes
*EC*: Effector cell
*EC*: Esophageal cancer
*ECM*: Extracellular matrix
*EMT*: Epithelial-mesenchymal transition
*FDR*: False discovery rate
*GC*: Gastric cancer
*GEO*: Gene Expression Omnibus
*GO*: Gene Ontology
*GS*: Gene significance
*GSEA*: Gene set enrichment analysis
*GSVA*: Gene set variation analysis
*HNSC*: Head and Neck squamous cell carcinoma
*ICB*: Immune checkpoint blockade
*IPS*: Immunophenoscore
*KEGG*: Kyoto Encyclopedia of Genes and Genomes
*KM*: Kaplan–Meier
*LOX*: Lysyl oxidase
*LOXL1*: Lysyl oxidase like 1
*MAF*: Mutation Annotation Format
*MDSC*: Myeloid-derived suppressor cells
*MM*: Module membership
*MSigDB*: Molecular Signatures Database
*NSCLC*: Non-small cell lung cancer
*OS*: Overall survival
*PCA*: Pancreatic cancer
*PDC*: Pancreatic ductal carcinoma
*pN stage*: Pathologic N stage
*PR*: Partial response
*RNA-Seq*: RNA-Sequencing
*SC*: Suppressor cells
*ssGSEA*: Single sample gene set enrichment analysis
*TCGA*: The Cancer Genome Atlas
*TIDE*: Tumor immune dysfunction and exclusion
*TILs*: Tumor-infiltrating lymphocytes
*TMB*: Tumor mutation burden
*TME*: Tumor microenvironment
*TOM*: Topological overlap matrix
*TPM*: Transcripts per kilobase million
*WGCNA*: Weighted gene co-expression network analysis
