# Supplementary material for: LOXL1 promotes tumor cell malignancy and restricts CD8 + T cell infiltration in colorectal cancer
Source: Cell Biol Toxicol. 2024 Jan 25;40(1):6. doi: 10.1007/s10565-024-09840-1 (PMC10808464; doi:10.1007/s10565-024-09840-1)
Supplement: Supplementary file 1 — Supplementary file1 (DOCX 17 KB) [file 10565_2024_9840_MOESM1_ESM.docx]

**Supplementary Figure 1. Identification of hub gene by WGCNA.**

**(A)** The sample clustering tree of CRC samples in the GSE39582 discovery cohort. The color band in the lower panel represents the age, gender, pathological T, N stage and CD8+ T cell infiltration level of patients. **(B)** Determination of the optimal soft-thresholding power in WGCNA. **(C)** Gene clustering dendrograms with gene dissimilarities based on topological overlap labelled with assigned module colors. Each branch in the figure represents one gene; each color indicates a single module that contains weighted co-expressed genes. **(D)** A scatterplot showing the correlation of gene significance for CD8+ T cell versus module membership in the turquoise module. **(E)** A scatterplot showing the correlation of gene significance for pathological N stage versus module membership in the turquoise module.

**Supplementary Figure 2. Functional enrichment analysis based on DEGs.**

**(A)** GO analysis was performed for the DEGs and significantly enriched terms were demonstrated in a circle plot (left panel) and table (right panel). **(B)** KEGG analysis for the DEGs indicating the enriched pathways through a cnetplot.

**Supplementary Figure 3. Mutation characterization of low and high LOXL1 expression cohort.**

**(A)** Kaplan-Meier curves for the OS of TCGA-COAD patients stratified by tumor mutation burden (TMB) level. **(B)** Stacked bar plot showed that the expression of LOXL1 was significantly associated with high TMB (Chi-square test, *P* = 0.018). **(C)** Comparison of TMB in the high and low LOXL1 expression groups. The Student’s *t* test was used for statistical analysis. **(D)** The scatterplot depicted the positive correlation between LOXL1 expression and TMB (The Pearson’s correlation coefficient R = 0.11, *P* = 0.026). **(E)** The variant classification, variant type and single nucleotide variant (SNV) class were summarized by different categories, and the top 10 mutated genes were displayed. **(F)** The percentage of mutations in different SNV types. **(G)** The heatmap showed the mutual co-occurring and exclusive mutations of the top 25 frequently mutated genes. The color of each cell represents the statistical significance of the exclusivity or co-occurrence of each pair of genes. Asterisks indicate statistical *P*-values <0.05, and points indicate statistical *P*-values <0.1. **(H, I)** Oncoplot displaying the tumor somatic mutation landscape of the 20 top genes with the highest mutation frequency in the LOXL1-high expression group **(H)** and LOXL1-low expression group **(I)**. The bar chart on the right panel represented the mutational types and the mutation percentage of each gene was calculated. The total number of mutations of each patient was shown on the upper panel.

**Supplementary Figure 4. Elevated LOXL1 Expression in Tumors Across Multiple Databases.**

**(A)** Differential expression of LOXL1 in colorectal cancer (CRC) versus normal tissues, as depicted in the UALCAN portal using TCGA data. **(B)** Pan-cancer analysis of LOXL1 expression levels utilizing the TIMER 2.0 database. **(C, D)** Evaluation of LOXL1 expression in CRC using the TNM plot database, with **(C)** focusing on RNA-Seq data and **(D)** on Gene chip data.

**Supplementary Figure 5. The expression and mutation landscape of LOXL1 in pan-cancer.**

**(A)** The expression levels of LOXL1 in normal, tumor, and metastatic tissues of BRCA, COAD, KIRC, LIHC, LUAD, OCSCC, OV and PAAD based on TNM plotter database. **(B)** The expression level of LOXL1 were analyzed in different pathological stages of BRCA, CESC, KIRC, LUAD, STAD and THCA from TCGA dataset. **(C)** The upper panel showed the disease-free survival (DFS) map using the online tool of GEPIA2. The Kaplan–Meier survival plots in the lower panel indicated that high LOXL1 expression correlated with poor DFS in different kinds of cancer (COAD, GBM KIRC, LGG and STAD). **(D)** The genetic alteration landscape of LOXL1 in TCGA pan-cancer from cBioportal database. **(E)** The mutation sites of LOXL1 in pan-cancer.
