# Supplementary material for: LOXL1 promotes tumor cell malignancy and restricts CD8 + T cell infiltration in colorectal cancer
Source: Cell Biol Toxicol. 2024 Jan 25;40(1):6. doi: 10.1007/s10565-024-09840-1 (PMC10808464; doi:10.1007/s10565-024-09840-1)
Supplement: Supplementary file 9 — Supplementary file9 (DOCX 14 KB) [file 10565_2024_9840_MOESM9_ESM.docx]

| **Table S8. Clinicopathological characteristics of the TCGA cohort in the *LOXL1* high and low groups** | | | |
| --- | --- | --- | --- |
| **Variables** | ***LOXL1*-high**  **(n = 226)** | ***LOXL1*-low**  **(n = 226)** | ***P-*value** |
| Age |  |  |  |
| ≥ 65 | 143(63%) | 137(61%) | 0.628 |
| < 65 | 83(37%) | 89(39%) |  |
| Gender |  |  |  |
| Female | 121(54%) | 93(41%) | **0.011** |
| Male | 105(46%) | 133(59%) |  |
| Stage |  |  |  |
| Stage Ⅰ | 35(16%) | 40(18%) | 0.4138 |
| Stage Ⅱ | 87(39%) | 87(40%) |  |
| Stage Ⅲ | 72(32%) | 56(26%) |  |
| Stage Ⅳ | 30(13%) | 35(16%) |  |
| Histological type |  |  | **0.004** |
| Adenocarcinoma | 181(81%) | 205(91%) |  |
| Mucinous Adenocarcinoma | 42(19%) | 20(9%) |  |
| Survival status |  |  | 0.498 |
| Alive | 172(76%) | 179(79%) |  |
| Dead | 54(24%) | 47(21%) |  |
| OS |  |  | **<0.001** |
| <365 Days | 202(89%) | 145(64%) |  |
| 365-1095 Days | 21(9%) | 39(17%) |  |
| >1095 Days | 3(1%)  (40%) | 42(19%) |  |
| Neoadjuvant therapy |  |  | 1 |
| No | 224(99%) | 225(99%) |  |
| Yes | 2(1%) | 1(1%) |  |
| Radiation therapy |  |  | 0.5995 |
| No | 220(97%) | 217(96%) |  |
| Yes | 6(3%) | 9(4%) |  |
